# siRNA Therapeutics: From Bench Lab. to Clinics

**DOI:** 10.3390/ph17040416

**Published:** 2024-03-26

**Authors:** Cristina Romero-López, Alfredo Berzal-Herranz

**Affiliations:** Instituto de Parasitología y Biomedicina López-Neyra, CSIC, PTS Granada, Av del Conocimiento 17, 18016 Granada, Spain

The discovery of the RNA interference (RNAi) mechanism in 1998 by Andrew Fire and Craig C. Mello [1] entailed an important breakthrough in the understanding of gene expression regulation at the post-transcriptional level mediated by RNA. Far from being a sporadic phenomenon, RNAi underlies the control of important cellular pathways in most living organisms. From a general point of view, RNAi can be defined as the process by which small interfering RNAs (siRNAs) and microRNAs (miRNAs) induce gene-specific silencing, either through the degradation of the target RNA or the downregulation of translation, respectively. siRNAs and miRNAs are short RNA duplexes, ranging from 21 to 23 nucleotides long in the case of siRNAs and 19 to 25 nucleotides long for miRNAs and ending in a two-nucleotide 3′ overhang. Both siRNAs and miRNAs derive from dsRNAs that are processed by RNase III enzymes and loaded into the RNA-induced silencing complex (RISC) to promote gene expression control [2,3]. However, while both types of molecules are structurally and functionally similar, there exist important differences between them [3]:

1. Regarding biogenesis, siRNAs regulate the genes from which they are expressed, whereas miRNAs are encoded by a gene other than their target gene.
2. siRNAs require full complementarity with the target RNA; thus, a single siRNA can induce silencing of a single target RNA, at least theoretically. Meanwhile, a single miRNA can regulate different genes since it does not require full complementarity with the target.
3. siRNAs are produced in organisms that lack a cellular immune response; their presence in mammals is currently unknown.

Long dsRNAs were successfully used for triggering RNAi response in plants, nematodes and Drosophila melanogaster. However, this strategy seemed limited in mammalian cells since long dsRNA molecules induce the interferon response pathway. Nevertheless, in 2001, two independent groups led by Tuschl [4] and Caplen [5] demonstrated that synthetic siRNAs, mimicking those naturally produced in nematodes, were able to trigger RNAi response in mammalian cells [4]. These findings revealed the potential of RNAi for disease treatment, leading to the development and implementation of RNAi tools [3]. Currently, it is accepted that RNAi-based therapies can be accomplished by siRNAs and miRNAs [6]. Furthermore, since miRNAs have multiple targets, their use as biomarkers and diagnostic agents has been widely investigated [7].

In the last two decades, works in different areas have evidenced the limitations of the efficient use of siRNAs and miRNAs as therapeutic tools, such as low bioavailability, cell delivery, instability, polyanionic composition and different unintended side effects, such as off-target inhibition, saturation of the RNAi machinery or cellular response activation [8,9]. Finally, the target accessibility must be taken into account when designing efficient siRNA and miRNA tools. For that purpose, it can be useful to perform both in silico and in vitro structural analyses with the aim of detecting regions within the target molecule that may be easily accessible (contribution 1). Identification of these drawbacks has been critical for the emergence of second-generation siRNA and miRNA molecules. The incorporation of chemical modifications has been of great interest since they have implemented the use of siRNAs and miRNAs as pharmaceutical drugs and biotechnological tools [10,11]. 

One of the major challenges for the use of siRNA-based therapeutics concerns the proper delivery to the target cells [12]. This aspect is highlighted in the review articles presented in this Special Issue by Missailidis and coworkers (contribution 2) and by Grassi and coworkers (contribution 3). In the first example, a historical review of the discovery of the interference phenomenon and its potential use as a biotechnological and therapeutic tool is given. In addition to providing a description of the mechanism of action of siRNAs, the manuscript by de Brito e Cunha et al. delves into the design of siRNAs and their internalisation into the target cell, reviewing the work carried out in the field of chemical modifications to produce more efficient, low-saturating siRNAs with improved pharmacokinetic and pharmacodynamic properties. The authors also provide an exhaustive review of the clinical trials that were initiated with siRNAs, placing special emphasis on those that have rendered commercially available drugs dedicated to the treatment of metabolic or rare diseases. Together with oncological processes, these diseases have been the main focus of pharmaceutical companies when developing new treatments based on siRNAs, as highlighted by the authors. Finally, the article presents a good summary of the pros and cons of using siRNA-based therapies.

Gabriele Grassi and coworkers also provide an interesting overview of siRNA therapeutics, with special emphasis on the application of these technologies for the treatment of gastrointestinal tumours (contribution 3), such as gastric, hepatocellular, pancreatic and colorectal cancer. They discuss those research works focused on the development of siRNAs aimed at controlling liver fibrosis and cirrhosis since both of them are risk factors for hepatocellular carcinoma. In their article, the authors provide an overview of siRNAs, their chemical features and function. The extensive discussion provided about the different delivery systems to the target cell is remarkable, highlighting the advantages and shortcomings of each of the approaches developed to date. Finally, the authors describe ongoing clinical trials aimed at testing siRNA-based therapeutics targeting different types of gastrointestinal tumours. The authors also highlight the difficulty of developing these studies due to the variability of the tumours to be treated, their dispersion throughout the patient’s body and the fact that, in some patients, the tumours multiply. Once again, the authors pay special emphasis on the delivery systems developed to formulate these new compounds.

Following the efficient siRNA delivery, this Special Issue includes an interesting research article by Anna Egorova et al. (contribution 4). In this work, they proposed a combinatorial approach by generating a therapeutic cocktail composed of three siRNAs targeting the mRNAs encoding the factors AQP3, CDC20 and COL4A2, all of them related to cell proliferation and metastasis in triple-negative breast cancer (TNBC), one of the most aggressive and difficult-to-treat tumours [13]. Taking advantage of previous studies with a peptide ligand targeting the CXCR4 receptor (the so-called L1 carrier) [14,15], the authors produced L1–siRNA conjugates with improved cell internalisation and low cytotoxicity, even at low cell density. The combination of conjugates with different target specificity rendered a more efficient drug, leading to significant inhibition of cell mobility and proliferation in breast cancer cells. These promising results open a new field of study using non-viral vehicles for siRNA-based therapeutics, not only in TNBC but also in solid tumours.

Related to the treatment of solid malignant tumours, Dr. Galera and coworkers present a potentially therapeutic strategy based on the use of miRNAs (contribution 5). It is worth noting the versatility of the use of miRNAs as therapeutic agents since they can be used, on the one hand, as replacement molecules in diseases produced by a deficit of the miRNA in question, as demonstrated by the use of miR-34a in different types of osteosarcoma [16], or as gene expression inhibitors. This last strategy was exploited by Dr. Galera and coworkers to identify five miRNAs that stimulate apoptosis in a chondrosarcoma cell line by targeting mRNAs required for cellular proliferation [17]. The obtained results led the research group to perform an interesting work that is included in this Special Issue. In their article, the authors extend their previous findings from chondrosarcoma to osteosarcoma cell lines in order to validate the specificity of the selected miRNAs. The results showed that two of the five miRNAs induced cytotoxicity in three different osteosarcoma cell lines (contribution 5). Furthermore, authors found that the key signalling pathway associated with these miRNAs was preserved both in chondrosarcoma and osteosarcoma cell lines, suggesting the potential of these molecules as useful drugs for the treatment of different bone-related tumours.

miRNAs play their function in the cell in which they are produced, but also, like other non-coding RNAs (ncRNAs), they participate in cell-to-cell communication via their exocytosis as extracellular vesicles (EVs), which can go through different physiological barriers [18,19]. The miRNAs’ incorporation into these vesicles increases their stability and their specific transport to the target cell, allowing them to be considered useful drugs for future therapies for difficult-to-treat diseases. This is the case of pulmonary fibrosis, a chronic lung disease with increasing prevalence in Europe and North America. The review article by Li et al. (contribution 6) offers a complete review of studies that have been carried out with EV extracted from different sources in animal models, emphasizing the potential role of EV-carrying therapeutic miRNAs as a candidate tool for treating pulmonary fibrosis. Furthermore, it provides a very enlightening discussion about the role of macrophage-based therapy as a lung-repairing treatment.

As it has been mentioned above, an additional advantage of miRNAs is that they can be used as biomarkers, particularly in many types of cancer that frequently show up- or downregulation of different miRNAs. The research article by Papagiannopoulos et al. (contribution 7) describes the identification of miRNAs that can be exploited as biomarkers of erythroleukemia, a subtype of chronic myeloid leukaemia caused by hyperproliferation of erythroid precursors. The authors found that several miRNAs were upregulated in differentiated erythroid cells, among them miR-16-5p, which had been previously shown to interfere with the proliferation rates of breast cancer cells [20,21]. They demonstrate that the expression of different factors related to ribosome biogenesis can be affected by miR-16-5p, leading to a misbalance of the overall ribosome levels in the cellular cytoplasm. Thus, this work shows a plausible mechanism for the observed antitumoral function of miR-16-5p and points to it as an interesting candidate for the treatment of different erythroid disorders. 

In summary, in this Special Issue, readers will find review articles that will provide them with general information about the use of RNAi technology in clinical and biomedical applications. Readers will also find research papers that will serve as clear and relevant examples of the potential of these molecules for the development of new therapeutic approaches and personalised medicine.
